# Comparative evaluation of the cytotoxic effect of two different gutta percha solvents

**DOI:** 10.1038/s41598-025-21759-z

**Published:** 2025-10-17

**Authors:** Esraa Samir S. Faisal, Mohamed I. Rabie, Sherouk H. Hassan Hussein

**Affiliations:** 1 Department of Endodontics, Faculty of Dentistry, Sinai University, Sinai, Egypt; 2 Department of Endodontics, Faculty of Dentistry, Suez Canal University, Ismailia, Egypt

**Keywords:** Cytotoxicity, Grapefruit oil, Orange oil, WST-1 assay, Gutta percha solvents, Biotechnology, Drug discovery, Medical research

## Abstract

Gutta percha solvents play an important role in removing filling materials from dentinal tubules, allowing irrigation solution to penetrate the tubules. The solvents should be biocompatible and have minimal effects on the viability of periapical tissues. Otherwise, they will result in intense inflammatory reactions and interfere with periapical healing. Aim This in vitro study was to evaluate and compare the cytotoxicity of two gutta percha solvents; grapefruit oil and orange oil. Biological testing was carried out on human fibroblasts that were retrieved from the cell bank and then cultured. Two gutta percha solvents (grapefruit oil and orange oil) were added to the cultured cells. Cell viability was evaluated using a WST-1 assay. The effect was evaluated after exposure to various concentrations at 24 h and 72 h. Statistical analysis was performed using two-way analysis of variance (ANOVA). Grapefruit oil had the most significant cytotoxicity followed by orange oil which had the least significant cytotoxicity. The cytotoxicity of the two solvents used was directly proportional to their concentration. Orange oil could be recommended as a solvent for gutta percha because of its low toxicity.

## Introduction

Successful root canal retreatment depends mainly on the complete eradication of the filling materials of the old root canal and the cleaning of any remnants that are responsible for endodontic failure^1^. The use of solvents is commonly recommended. They can access and dissolve filling materials and reach the dentinal tubules and anatomical ramifications without creating mechanical errors^2^. In retreatment procedures without using solvents, mechanical damage to the original endodontic space can take place^3^.

The solvents should be biocompatible, have minimal effects on the cell viability of the periapical tissues, and be free of any hazards^4^. Otherwise, they will result in intense inflammatory reactions, which in turn can induce tissue destruction and interfere with the peri-radicular healing^5^. The biocompatibility of these gutta-percha solvents can be evaluated by many parameters, including in vitro cytotoxicity tests. WST-1 assay (Water-soluble tetrazolium salt) is a colorimetric test for assessing cell viability and cytotoxicity. The principle of this assay is based on the transformation of the tetrazolium salt into a highly water-soluble formazan by mitochondrial dehydrogenase enzymes in the presence of an intermediate electron acceptor, such as mPMS (1-methoxy-5-methyl-phenazinium methyl sulfate). Water-soluble salts are released into the cell culture medium. Within the incubation period, the reaction produces a color change that is directly proportional to the amount of mitochondrial dehydrogenase in the cell culture; thus, the assay measures the metabolic activity of cells^6^. Several synthetic chemical solvents such as chloroform and xylene, are most commonly used in endodontic retreatment; however, their use is restricted because of their carcinogenic potential and cytotoxicity^7^. This has encouraged researchers to look for herbal alternatives. The use of alternative solvents such as essential oils in endodontics is increasing because of their proven biocompatibility, low toxicity, and noncarcinogenicity. Some of them have been reported as gutta-percha solvents, such as orange oil and grapefruit oil. Orange oil is one of the most widely used gutta-percha solvents in endodontic retreatment. It is ideal for rapid opening of root canals and softens gutta-percha cones during endodontic retreatment^8^. Grapefruit (Citrus paradisi) is one of the largest products of citrus families and is extracted from the grapefruit peel. During penetration into hard-to-reach places, grapefruit oil is better than other solvents washed out of them, since it has the best wettability for irrigation solutions^9^.

Recently, grapefruit oil has been introduced as a new endodontic solvent and has been shown to effectively dissolve gutta percha and to be more effective than other essential oils^10^. Thus, this research aimed to compare the cytotoxicity of grapefruit oil and orange oil by using WST-1 assay. The null hypothesis of the study was that there is no significant difference between grapefruit oil and orange oil regarding cytotoxicity.

## Methods

### Study setting and ethical considerations

The study procedures received approval from the Research Ethics Committee (REC) of the Faculty of Dentistry, Suez Canal University, under authorization number 435/2021, in accordance with the Helsinki Declaration of the World Medical Association (2008 Version).

### Specimen preparation and grouping

The gutta percha solvents used for this study were grapefruit oil and orange oil. The two gutta percha solvents were initially prepared as a 50 mg/ml stock. For the dose-response curve, the stock solutions were diluted in cell culture medium (Dulbecco’s modified Eagle’s medium) to achieve a total of six concentrations (0.01, 0.1, 1, 10, 100 and 1000 mg/mL) (Table 1).

According to sample size calculations, a total sample size of 96 wells were used, in which each concentration (M1, M2, M3, M4, M5 and M6) of each solvent (A, B) and control group (C) was used (Table 2).

**Table 1:** Variables of the study and levels of investigation for WST-1.

Variable	Level	Description
Type of Gutta percha solvent	A	Group (A), Grapefruit oil Cells grown in medium conditioned by Grapefruit oil
	B	Group (B), Orange oil Cells grown in medium conditioned by Orange oil
	C	Group (C), Control group Cells grown in fresh medium
Concentration (M)	M_1_	0.01
	M_2_	0.1
	M_3_	1
	M_4_	10
	M_5_	100
	M_6_	1000

**Table 2:** Study design of WST-1 (*n* = 3).

Variables time interval					Concentrations (M)										Total
					Follow-up										
		**T**_**1**_ **(after 24 h)**						**T**_**2**_ **(after 72 h)**							
		**M**_**1**_	**M**_**2**_	**M**_**3**_		**M**_**4**_	**M**_**5**_	**M**_**6**_	**M**_**1**_	**M**_**2**_	**M**_**3**_	**M**_**4**_	**M**_**5**_ **M**_**6**_		
Types of gutta percha solvent	**G-A**	AM_1_	AM_2_	AM_3_		AM_4_	AM_5_	AM_6_	AM_1_	AM_2_	AM_3_	AM_4_	AM_5_ AM_6_		**36**
		**3 wells**	**3 wells**	**3 wells**		**3 wells**	**3 wells**	**3 wells**	**Repeat for all**						
	**G-B**	BM_1_	BM_2_	BM_3_		BM_4_	BM_5_	BM_6_	BM_1_	BM_2_	BM_3_	BM_4_	BM_5_ BM_6_		**36**
		**3 wells**	**3 wells**	**3 wells**		**3 wells**	**3 wells**	**3 wells**	**Repeat for all.**						
	**G-C**		No solvent **(18 wells)**						**(6 blank)**						**24**
Total samples															**96**

### Cell culture

Human fibroblast cells were retrieved from the cell bank (ATCC). The cells were maintained in complete growth medium containing Dulbecco’s modified Eagle’s medium (DMEM high glucose) supplemented with 100 µg/ml of streptomycin 100 units/ml penicillin and 10% of heat-inactivated fetal bovine serum (FBS) (Sigma, Seelze, Germany). The cells were preserved in an incubator (Thermo Fisher Scientific, Waltham, USA) at 37 °C in humidified air and 5% CO2 for 1 h. The cells were cultured in plastic tissue culture dishes. The cell culture medium was changed every 2 or 3 days until the cells reached 80% confluence for optimal cell harvesting, after which the cells were detached with 0.5% (w/v) trypsin-EDTA for subculturing. The growing cells were diluted in fresh medium and seeded into 96-well plates (3 × 10^3^ cells/well). Cell seeding density was optimized within the range of 1–10 × 10³ cells/well to ensure continuous exponential growth and to avoid early confluency during the experimental period. All steps were carried out under aseptic conditions (vertical cleanroom workbench BIO-CL).

### Cytotoxicity assay (WST-1)

**Fig. 1:**
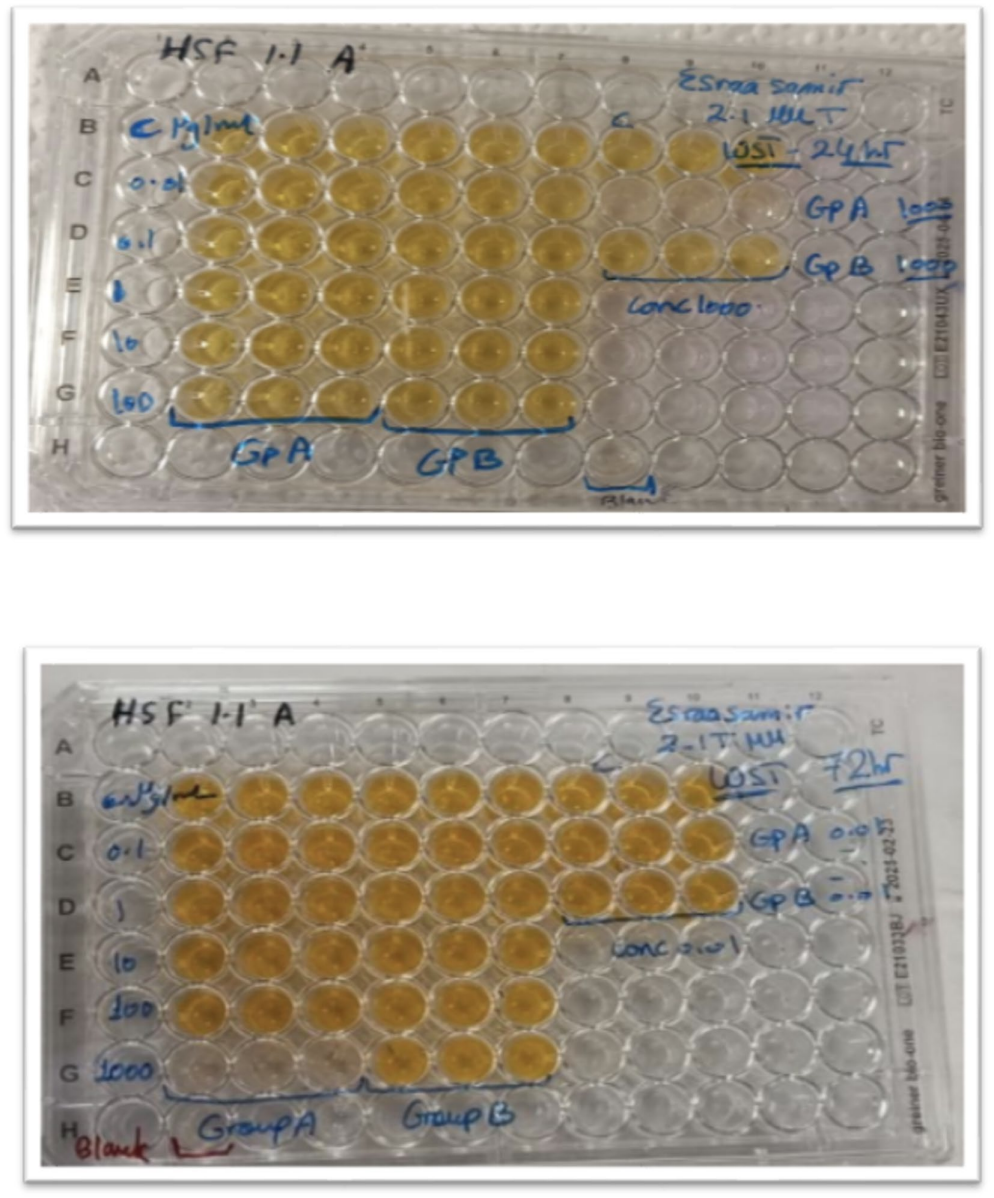
A photograph showing the reduction in intracellular soluble yellow tetrazolium (WST-1 dye) after 24 and 72 h.

Cell viability was assessed by a WST-1 assay using an Abcam kit (ab155902 WST-1 Cell Proliferation Reagent). Cultured cells in 96-well plates in 200 µl of media (except for 6 wells which were left empty – blank controls) were incubated at 37 °C in air containing 5% CO2 and at 95% relative humidity for 24 h. After incubation for 24 h, the medium was aspirated from all wells and replaced blindly with other aliquots of 50 µL/well of serial dilutions of the used gutta percha solvents (0.01, 0.1, 1, 10, 100 and 1000) or control medium. After 24 h of solvent exposure, the cells were treated with 10 µL of WST-1 reagent. The cells were incubated for another 72 h for evaluation by the WST-1 assay (Fig. 1). and the absorbance at 450 nm was measured after 1 h using a microplate reader (FLUOstar Omega microplate reader).

The average absorbance of blanks (WST-1 without cells) was subtracted from sample absorbances and absorbances were corrected by their respective reference. The corrected absorbance of the dead cells was subtracted from the corrected live cells absorbance. The results are expressed relative to the control.

Assay was performed in triplicate to ensure reproducibility. The absorption value obtained with the untreated fibroblasts as a control was deemed to indicate 100% viability.

**Cell Viability Classification**:

The classification of cytotoxicity was performed according to the ISO 10993-5:2009. Percentage of Viable Cells = (A/B) × 100 where A = viable cells in the experimental well and B = viable cells in the control well. A cell viability greater than 90% is considered as noncytotoxic, 60% to 90% is considered slightly cytotoxic, 30% − 59% is considered cytotoxic and less than 30% cell viability is considered strongly cytotoxic.

### Scanning electron microscope (SEM) analysis

Cell morphology was assessed using scanning electron microscopy (SEM). After exposure to the tested material at different concentrations and time intervals (24 h and 72 h), the cells were fixed with 2.5% glutaraldehyde in 0.1 M phosphate buffer (pH 7.4) for 2 h at 4 °C. Samples were then washed with phosphate buffer and post-fixed in 1% osmium tetroxide for 1 h. Dehydration was performed followed by drying with hexamethyldisilazane. Finally, samples were sputter-coated with a thin layer of gold and examined under a scanning electron microscope (Model: [SEM-EDS System], Manufacturer: [JEOL, Japan]) at an accelerating voltage of 15 kV.

### Statistical analysis

Data were calculated, tabulated, and statistically analyzed. A normality test (Kolmogorov-Smirnov) was done to check normal distribution of the samples. Statistical analysis was performed using SPSS software for windows version 22.0 (IBM Corp Armonk, NY, USA) at significant levels of 0.05 (P- Value ≤ 0.05). Descriptive statistics were calculated as Mean ± Standard deviation (SD), range (Minimum- Maximum). In the statistical comparison between the different groups, the significance of difference was tested using two-way ANOVA (analysis of variance) to compare between the two factors (type of solvent & concentrations) and their interactions on cytotoxicity. Tukey`s post hoc test was performed for the evaluation of statistical significances among the groups. P value < 0.05 is considered be statistically significant.

## Results

### Comparison between the two gutta percha solvents at different concentrations (intergroup analysis)

#### After 24-hour intervals

**Fig. 2:**
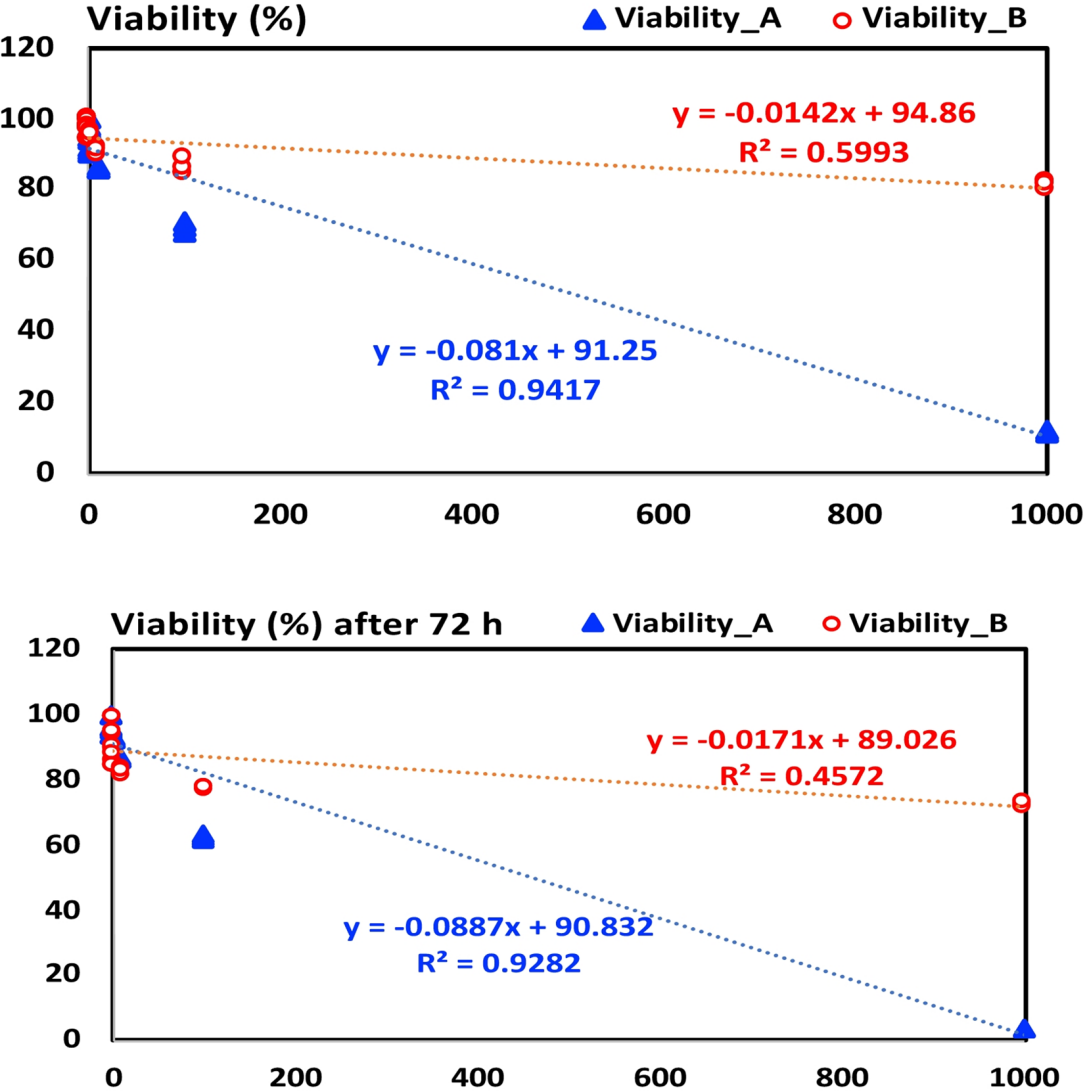
Line curve showing comparison of the change in viability (%) (Y-axis) with increasing concentration (X-axis) after 24 and 72 h.

There was a statistically significant difference between group A (grapefruit oil) and group B (orange oil) at different concentrations (0.01, 1, 10, 100, and 1000). However, at 0 and 0.1. the difference between A and B was not significant. The cytotoxicity of the two solvents was directly proportional to the concentration. (Table 3; Fig. 2).

**Table 3:** Comparison between cytotoxicity (as viability %) of the two gutta percha solvents at various concentrations after 24 h. Data presented as mean ± SE.

Concentration	Viability after 24 h/Groups						Independent t-test
	A			B			
	Mean	SD	DMRTs	Mean	SD	DMRTs	
0	**100.0**	0.00	a	**100.0**	0.00	a	> 0.05 ns
0.01	**95.8**	1.45	c	**98.2**	0.81	ab	0.034*
0.1	**95.4**	1.83	c	**96.1**	1.94	bc	0.666 ns
1	**91.0**	0.84	d	**95.2**	1.18	c	0.007**
10	**85.8**	0.39	e	**91.0**	1.19	d	0.002**
100	**69.2**	1.24	g	**86.4**	2.16	e	< 0.001***
1000	**11.7**	0.64	h	**81.4**	1.35	f	< 0.001***
ANOVA (p-value)	< 0.001***			< 0.001***			
Correlation	*r*= −0.972; *p* < 0.001***			*r*= −0.774; *p* < 0.0001***			
Regression R2	0.974; *p* < 0.001***			0.599; *p* < 0.001***			

#### After 72-hour intervals

There was a statistically significant difference between group A (grapefruit oil) and group B (orange oil) at different concentrations from (0.1, 1, 10, 100, and 1000). However, at 0 and 0.01. the difference between group A and group B was not significant. The cytotoxicity of the two solvents was directly proportional to the concentration. (Table 4; Fig. 2).

**Table 4:** Comparison between cytotoxicity (as viability %) of the two gutta percha solvents at various concentrations after 72 h. Data presented as mean ± SE.

Concentration	Viability (%) after 72 h/group						Independent t-test
	A			B			
	Mean	SD	DMRTs	Mean	SD	DMRTs	
0	**100.0**	0.00	a	**100.0**	0.00	a	> 0.05 ns
0.01	**95.6**	0.92	b	**94.9**	0.21	b	0.258 ns
0.1	**94.8**	0.79	b	**89.1**	2.62	d	0.023*
1	**92.9**	0.71	c	**86.1**	1.88	e	0.004**
10	**87.2**	0.47	e	**83.2**	0.97	f	0.003**
100	**62.8**	0.83	i	**78.0**	0.44	g	< 0.001***
1000	**4.1**	0.28	j	**72.9**	0.69	h	< 0.001***
ANOVA (p-value)	< 0.001***			< 0.001***			
Correlation	*r*= −0.988; *p* < 0.001***			*r*= −0.988; *p* < 0.0001***			
Regression R2	0.928; *p* < 0.001***			0.457; *p* < 0.001***			

### Comparison between different concentrations of each gutta percha solvent (intragroup analysis)

**Fig. 3:**
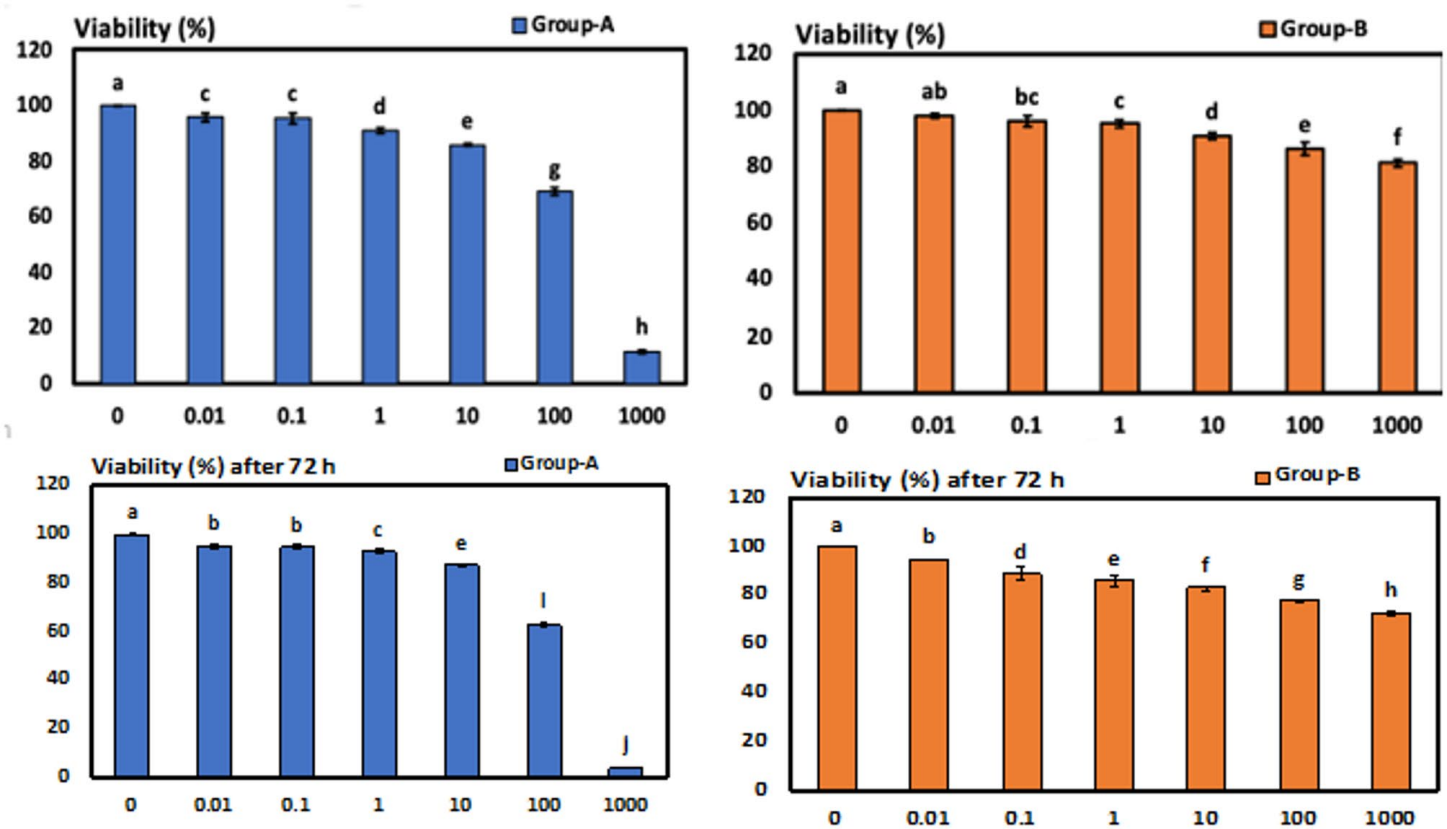
Chart presenting the intra group comparison between concentrations within Group (A) Grapefruit oil and Group (B) Orange oil after 24 and 72 h. Error bars represent mean ± SD.

The difference in viability (%) in group A (grapefruit oil) between concentrations was highly significant (< 0.001***). Additionally, the difference in viability (%) in group B (orange oil) between concentrations (intragroup) was highly significant (< 0.001***). (Fig. 3).

### Comparison of IC_50_ values (µg/mL) for grapefruit oil (Group A) and orange oil (Group B) at different exposure times

The IC50 values for Grapefruit oil (Group A) were 183.5 µg/mL at 24 h and 193 µg/mL at 72 h, indicating a concentration-dependent decrease in cell viability. In contrast, Orange oil (Group B) exhibited an IC50 greater than 1000 µg/mL at both time points, suggesting substantially lower cytotoxicity compared to Grapefruit oil.

### Scanning electron microscope (SEM) analysis

**Fig. 4:**
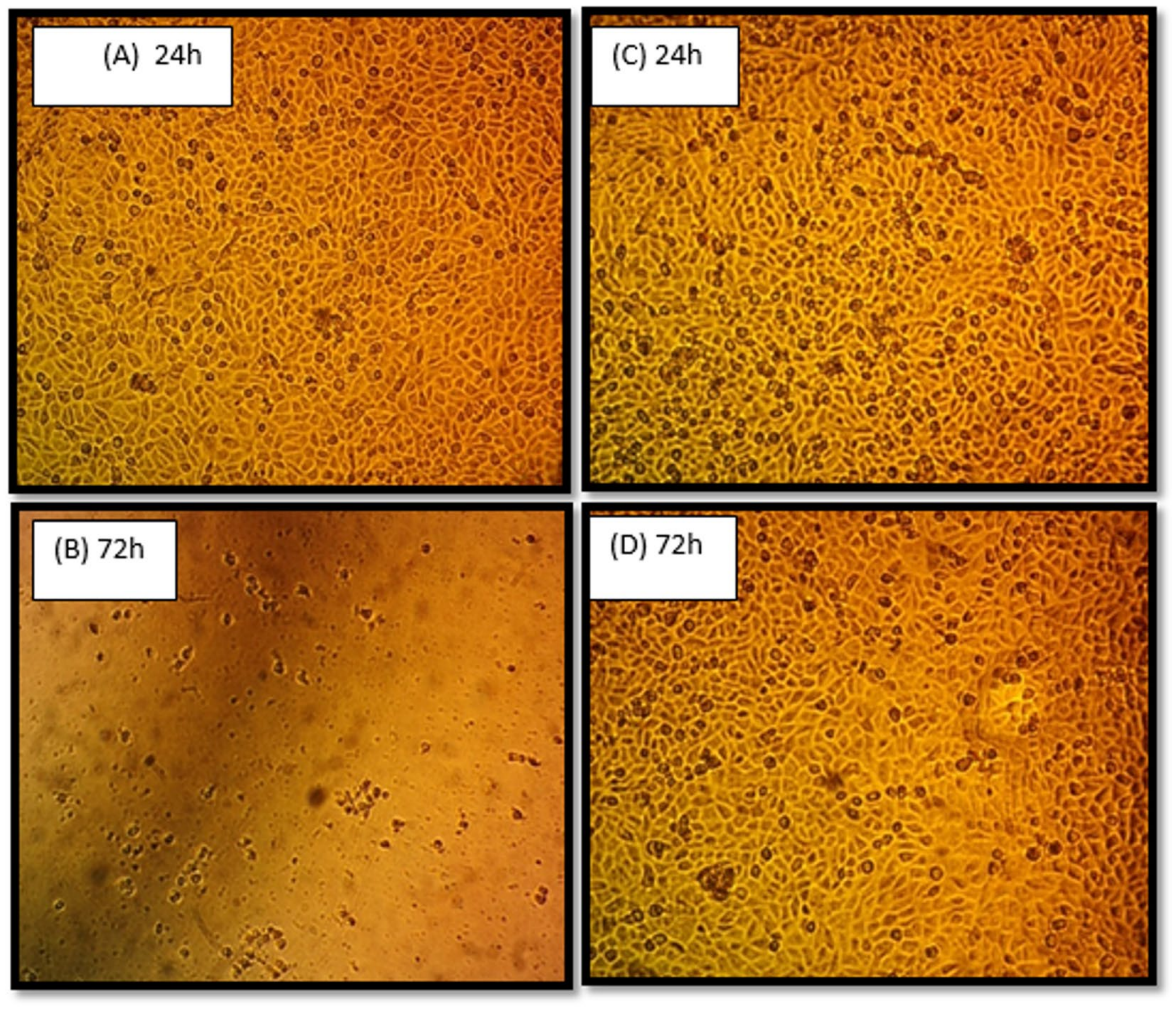
SEM microphotographs showing the viability of cells in Grapefruit oil (**A**) and (**B**) Orange oil (**C**) and (**D**) at different time intervals at 24 h and 72 h. Figure (**B**) shows that at conc 1000, cells were damaged due to shrinkage, round cells floating in the medium, and the presence of cell debris, which is specific for cytotoxicity, and cell death. Figure (**D**) shows that at conc 1000, There were significant changes in cell morphology such as the area of necrosis and the presence of some cell debris. (**C**) 24 h, (**D**) 72 h.

At 24 h, most cells preserved their normal spindle-shaped morphology, with minimal changes observed in Group B (Orange oil). However, after 72 h, pronounced cytotoxic effects were evident. These morphological changes are indicative of severe cytotoxicity and cell death (Fig. 4).

## Discussion

Nonsurgical endodontic retreatment involves the complete removal of filling materials and microorganisms from the root canal through proper disinfection, shaping, and finally obturation of the root canal system^11^. Several techniques and materials have been applied for endodontic retreatment. However, access to the obturating material for its removal is the obstacle to retreatment especially when it is well condensed and resistant to instrument penetration, most critically in curved regions of the root where perforation is a risk^12^. In such cases, the use of solvents is commonly recommended^13^. They are safer than the other techniques because they can access and dissolve filling materials and reach anatomical ramifications without creating mechanical errors^2^. During solvent-free retreatment, there is a risk of mechanical damage to the original endodontic space^3^. Chloroform is among the most effective solvents for treating gutta-percha. However, due to its cytotoxicity and carcinogenic properties, alternative solvents have been investigated^14^. The use of essential oils as alternatives in endodontics is growing because of their proven, low toxicity, and non-carcinogenicity compared with organic solvents^15^. Orange oil is one of the most used essential oils^16^. Grapefruit oil is an essential oil that has recently been used as a solvent for gutta-percha. When using grapefruit, the percentage of developing an infectious inflammatory process in dentin is reduced^10^. Scelza et al.^6^ demonstrated that orange oil was found to be less toxic than eucalyptol, xylene, and chloroform. Moreover, Rehman et al.^17^, determined that the efficiency of orange oil solvent was similar to that of chloroform, and they recommended it as a suitable alternative. In the WST-1 assay, the percentages of viable cells after contact with both solvents at the concentrations of 0.01, 0.1, and 1 were approximately 96%, 95%, and 91%, respectively, for grapefruit oil and 98%, 96%, and 95%, respectively, for orange oil. This suggests that both solvents are potentially nontoxic. However, both solvents increased in toxicity and became slightly toxic to the target cells at concentrations of 10 and 100 mg/mL. Oyama et al. compared several solvents, including chloroform, eucalyptol, and orange oil, and found that the latter was the least toxic, corroborating our findings^18^.

The choice for solvent of different concentrations was performed to observe a possible dose-response relationship, as the extracts can be subjected to serial dilutions, allowing for the evaluation in a manner similar to the increased dilution occurring in vivo. Thus, it is hoped that the clinical response of periapical cellular populations can be better anticipated. The application time of tissue reaction was assessed at different time intervals (24 and 72 h) permitting the observation of histological responses during short and long-term periods. According to the data of incubation of WST-1, intragroup comparisons showed that the cytotoxicity of the two solvents was directly proportional to the concentrations. These cell viability results agreed with those of a study by Correa et al., in which a lime essential oil sample increased cell viability with decreasing concentration, and the concentration of 1000 mg/mL resulted in the lowest cell viability among all the tested groups^19^. SEM analysis provided qualitative evidence supporting the cytotoxic effect of grapefruit oil on cell morphology at higher concentrations and prolonged exposure times. At 24 h, most cells maintained their normal spindle-shaped morphology with slight alterations. However, after 72 h at 1000 µg/mL, marked morphological changes were evident, including cellular shrinkage and the presence of cell debris. These features are characteristic of apoptosis and necrosis, indicating severe cytotoxicity. Similar findings were reported by Tyagi and Malik^20^, where essential oils induced cell shrinkage and membrane disruption under SEM. Furthermore, for a better understanding of the cytotoxic effects of the tested materials on cell permeability, analysis of the mode of cell death (apoptosis/necrosis) via flow cytometry and fluorescence microscopy has been applied, as it allows easy, sensitive, and nondestructive analysis of changes in the structure of the cell membrane with the application of fluorescence methods. In this way, vital, apoptotic, and dead cells can be distinguished based on double-labeling with annexin V and PI^21^. Regarding the healthy apoptotic cells, the difference between the two solvents was highly significant where Orange oil showed higher viable cells than Grapefruit oil. On the other hand, the early and late apoptotic cells, the difference between the two solvents was non-significant. Although a positive control (e.g., DMSO or Triton X-100) was not included in this study, the primary aim was to assess the relative cytotoxicity of grapefruit oil and orange oil concentrations compared to untreated control cells rather than to establish absolute cytotoxicity thresholds. The absence of a chemical positive control does not compromise the interpretation of the relative differences among the tested concentrations. According to the results of the present study, grapefruit oil, which is expected to be less toxic, showed greater cytotoxicity than orange oil. We rejected the null hypothesis as there was significant difference between the cytotoxicity of the two tested solvents (P-value < 0.001).

In light of our findings, we suggest avoiding excessive use of solvents and rinsing the root canal thoroughly immediately after the procedures. Our results provide sufficient information to dentists to warn them of any gutta-percha solvent discharge from the apical foramen. It is important to note that the present study was conducted entirely in vitro, assessing cytotoxicity and cell death pathways under controlled laboratory conditions.

## Conclusion

In terms of the WST-1 assay, the cytotoxicity of the two solvents was directly proportional to the concentration, as the percentage of viable cells increased with decreasing the concentration. Additionally, the cytotoxicity of solvents was directly proportional to time. The cytotoxicity caused by solvents increased with increasing time.

## Limitations

The study was conducted under in vitro conditions, which may not fully mimic the complex environment of human tissues in vivo.

The absence of a positive control which is typically used to confirm assay performance.

This study evaluated only the in vitro cytotoxicity of two gutta-percha solvents and did not assess their efficacy in gutta-percha removal. Therefore, the findings cannot be directly translated to clinical recommendations.

## Recommendations

Future in vivo research is required to clarify the cellular events that take place in the periapical tissues when orange oil and grapefruit oil are used as gutta percha solvents.

Further studies in vivo are needed to shed light on the adequate concentrations of grapefruit oil and orange oil that have solving efficacy and not toxic to the host stem cells.

Future studies should investigate both the biological safety and the efficiency of gutta-percha removal to provide a more comprehensive assessment of their clinical applicability.

Our study recommends the use of gutta percha solvents in lower concentrations and special attention should be paid to the prevention of solvent extrusion, distinctively in cases with wide root canal orifices or root perforations.

## Data Availability

All data generated or analyzed during this study are available from the corresponding author upon request for academic and non-commercial use. No restrictions apply.
